# No Association between Personality and Candidate Gene Polymorphisms in a Wild Bird Population

**DOI:** 10.1371/journal.pone.0138439

**Published:** 2015-10-16

**Authors:** Hannah A. Edwards, Gabriela K. Hajduk, Gillian Durieux, Terry Burke, Hannah L. Dugdale

**Affiliations:** 1 Department of Animal and Plant Sciences, University of Sheffield, Sheffield, United Kingdom; 2 Groningen Institute for Evolutionary Life Sciences, University of Groningen, Groningen, The Netherlands; 3 Nature Seychelles, Mahe, Republic of Seychelles; Università della Tuscia, ITALY

## Abstract

Consistency of between-individual differences in behaviour or personality is a phenomenon in populations that can have ecological consequences and evolutionary potential. One way that behaviour can evolve is to have a genetic basis. Identifying the molecular genetic basis of personality could therefore provide insight into how and why such variation is maintained, particularly in natural populations. Previously identified candidate genes for personality in birds include the dopamine receptor D4 (*DRD4*), and serotonin transporter (*SERT*). Studies of wild bird populations have shown that exploratory and bold behaviours are associated with polymorphisms in both *DRD4* and *SERT*. Here we tested for polymorphisms in *DRD4* and *SERT* in the Seychelles warbler (*Acrocephalus sechellensis*) population on Cousin Island, Seychelles, and then investigated correlations between personality and polymorphisms in these genes. We found no genetic variation in *DRD4*, but identified four polymorphisms in *SERT* that clustered into five haplotypes. There was no correlation between bold or exploratory behaviours and *SERT* polymorphisms/haplotypes. The null result was not due to lack of power, and indicates that there was no association between these behaviours and variation in the candidate genes tested in this population. These null findings provide important data to facilitate representative future meta-analyses on candidate personality genes.

## Introduction

Between-individual differences in behaviour that are consistent through time/context are referred to as personality [1]. Personality can be heritable (the average heritability estimate across 209 studies was 0.26 [SE = 0.01, 2]), correlated across contexts and have the potential to influence how populations adapt and evolve [2]. Why personality persists in populations is hard to explain given the assumption that behaviour should be centred on an optimum strategy or co-existing evolutionary stable strategies [3]. It is thought that personality could be maintained if the fitness pay-offs associated with a behaviour were frequency dependent [4] or state-dependent [5,6]. Despite the large heritable component personality can have [7], the genetic loci underlying personality are relatively unknown. Identifying these genetic loci may therefore be pivotal to our understanding of why personality occurs, and its ecological and evolutionary significance.

The most promising candidate genes for human personality traits are the dopamine receptor D4 (*DRD4)*, involved in the mediation of the hormone dopamine in the dopaminergic system, and the serotonin transporter (*SERT)*, which controls the uptake of the hormone serotonin in the synaptic clefts of the neurones [8]. Although null results have been found in candidate gene association studies [8], variation in novelty-seeking behaviour in humans is generally associated with polymorphisms in *DRD4* [9] and low levels of novelty seeking behaviour have been recorded in *DRD4* deficient mice [10]. Additionally, variation in measures of anxiety related behaviour in humans has been associated with polymorphisms in *SERT* [11,12] and high levels of anxiety related behaviour and low levels of novelty seeking have been reported in *SERT* deficient mice [13,14].

It is important to investigate these associations in natural populations because, although human personality is not directly comparable to animal personality, cultural determinants of behaviour are thought to be less influential in natural animal populations and can provide insight into why individual variation exists [8]. In captive animal populations the controlled, artificial environment of the laboratory can alter the expression of, and selection on, genetic variation [15,16,17]. In one of the first non-human studies of a wild population, a single nucleotide polymorphism (SNP) in exon 3 of *DRD4* (*SNP830*) was identified and the genotype *SNP830T* correlated with faster exploratory behaviour in a novel environment in one of four populations of great tits, *Parus major* [18]. A similar association was found in a wild population of collared flycatchers (*Ficedula albicollis*), where *DRD4 SNP554* was linked with neophobia and *DRD4 SNP764* with risk-taking behaviour [19]. *DRD4 SNP449* in two wild populations of invasive yellow-crowned bishops (*Euplectes afer*) was also found to be positively correlated with neophobic behaviour [20]. More recently, in free-ranging Rhesus macaques (*Macaca mulatta*) *DRD4* polymorphisms have also been associated with bold behaviour [21]. Conflicting results have been found in association studies investigating *SERT* in wild populations. Individuals heterozygous for *SERT* at *SNP758* and *SNP988* were more prevalent in rural, less neophobic populations of wild blackbirds (*Turdus merula*) when compared to urban populations, between 23–45 km apart in the same country [22]. In contrast, free ranging Rhesus macaques did not show an association between their exploration of novel stressors and their *SERT* genotype (C.E. Fleener, personal communication).

Here we investigated whether polymorphisms in the candidate genes for exploratory and for bold behaviour [23], *DRD4* and *SERT*, are associated with variation in these respective behavioural traits in a wild population of Seychelles warblers (*Acrocephalus sechellensis*). The Cousin Island population of Seychelles warblers should prove to be a good model for such a study, because adult individuals exhibit innate predator recognition behaviour [24]. Here we test the prediction that between-individual variation in exploratory behaviour and bold behaviour is associated with polymorphisms and/or haplotype in *DRD4* and in *SERT*.

## Methods

### Ethics statement

Local ethical regulations and agreements were followed for fieldwork. Nature Seychelles permitted us to work on Cousin Island Nature Reserve. The Seychelles Department of Environment and the Seychelles Bureau of Standards authorized fieldwork and sampling (permit reference AO157).

### Study system

The Seychelles warbler is an endemic facultative cooperative breeding passerine that occurs on five islands within the Seychelles. Dominant breeding pairs are territorial and socially monogamous. Paternity is gained predominantly by dominant rather than subordinate males and around 44% of offspring have extra-group fathers [25,26]. Due to habitat saturation, individuals are forced to assume subordinate roles [27]. Dominance status was assigned to individuals in pairs that were observed in a territory within close proximity of one another, had frequent vocal interactions and mate guarded [28]. A subordinate status was assigned to single birds consistently seen in a territory interacting with group members but not engaging in dominant pair behaviour.

At the study site of Cousin Island (29 ha; 048200 S, 558400 E), during the winter (Jan-Feb) and summer (Jun-Sep) breeding seasons in 2010–15 the breeding status of each individual was identified, territories mapped and birds caught with mist nets, colour/BTO ringed if necessary, and a blood sample obtained from a brachial venipuncture. The blood sample was later used for pedigree analysis and molecular sexing [29]. This population has been individually monitored since the 1980s, providing a long-term dataset with accurate measurements of survival and fecundity due to the 0.92 probability of re-sighting individuals in their first two years of life and 0.98 probability of re-sighting adults [30,31].

### Personality assays

Exploration behaviour was assayed in an Oxygen 4 tent (L322 x W340 x H210 cm; Gelert Ltd Wigan). The tent contained three artificial trees and each tree had two branches that were 45 cm long (one attached at 95cm and one attached to the top of the trunk), and a trunk, 148 cm high. The number of flights, hops and total number of trees visited were recorded in five minutes [32] and every minute over twenty minutes for twenty individuals to test for acclimation to the novel environment (see S1 Table for break point analysis results). A flight denoted a transfer between branches on the same tree, between trees or between floor and trees, or any movement greater than a branch length, involving flapping of the wings. A hop was described as both feet off the ground with no wing flapping, either on the same branch or on the floor. The numbers of hops, flights and unique trees visited were totalled to give a measure of exploration [33].

Bold behaviour was tested two minutes after the exploration assay to allow for habituation to the novel environment of the tent. A novel pink toy attached to a tree branch (95cm long) was inserted and positioned in the centre of the tent [32]. We also conducted a control assay with the novel toy excluded. The order of the bold and control assays were randomised and measured two minutes apart. The behaviour score (summed number of hops, flights and trees visited) was higher in the novel object assay than the control assay (Wilcoxon signed rank test; V = 2145, p<0.001), such that the behavioural reaction resulted from the novel object and not the stick it was attached to. Behaviour scores in the novel object assay were therefore used as a measure of bold behaviour similar to other studies [34,35,36,37]. Both exploratory behaviour and bold behaviour are repeatable in this study species. Repeatability was calculated using a generalised linear model with the package MCMCglmm [38]. Exploration had a repeatability of 0.23 (CI: 0.08–0.37, n = 173 assayed once and 139 assayed more than once), bold behaviour of 0.26 (0.05–0.52, n = 120 assayed once and 57 assayed more than once) and the correlation coefficient between these two behaviours was 0.60 (0.01–0.79, n = 177) H.A. Edwards, unpublished data).

### Primer design

**Fig 1:**
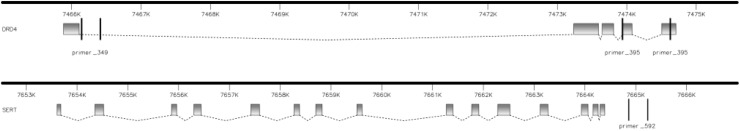
Schematic representation of the *DRD4* and *SERT* regions. Grey boxes represent exons and the dotted line introns. The vertical black lines indicate the locations of the primers used in this study.

*DRD4* exon 3 sequences from the great tit DQ006802, the chicken *Gallus gallus* NP001136321, blue tit (*Cyanistes caeruleus*) JN986724.1 and blackcap *Sylvia atricapilla* (AEC22814.1), and *SERT* chromosome 19 sequences from the blackbird *Turdus merula* KC584781, collared flycatcher *Ficedula albicollis* AGTO02004766.1 and zebra finch *Taeniopygia guttata* ABQF01026424, were aligned using Mega 5.2 [39] to design conserved primers. By looking for conserved sequences we designed suitable primers, tested their capability in Primer 3 0.4.0, length: 18–22 bp, melting temp: 59–61°C [40], and then ran the FASTA sequence in Genbank BLASTN 2.2.28 [41]. The graphical alignment output from BLASTN for the presence of conserved segments among the sequences was inspected to check that primer sets amplified the DNA products of predicted size and target area (Fig 1). Three primer sets resulted: DRD4_395 (709 bp of the end region), DRD4_349 (290 bp of the start region) and SERT_592 (394 bp of the non-coding end region, Table 1). Although SERT_592 amplified a non-coding end region (approx 470 bp from the end of *SERT* exon), non-coding regions can alter the level of gene expression and behaviour [42] and linkage disequilibrium (LD) was expected to be high in the Seychelles warbler.

**Table 1:** Sequence, melting temperature (T_m_) and length of product expected from each designed primer in base pairs.

Accession number	Primer ID	Primer sequence 5’-3’	T_m_ (°C)	Expected product length (bp)
LN833019	DRD4_395	F: GATATTCGCCTTTGCTGTGG	60.6	395
		R: TTCCTGAACTCGGCGTTG	60.6	395
LN833003	DRD4_349	F: CTCGCCCTCCTCGTCCT	60.6	349
		R: GACGGGGATCCCAGGAA	60.6	349
LN833076	SERT_592	F: TGGAACCACAGTGTCAGCAG	60.8	592
		R: CTGGATCACACCCTCTCAGG	60.8	592

### SNPs and genotyping

A power analysis [43] using the effect size in the Westerheide population from Korsten *et al*. [18] revealed that a sample size between 49–56 was sufficient to detect an effect of polymorphisms on behaviour (S2 Table). Fifty-seven individuals with repeat exploratory assays that belonged in the upper (n = 29) and lower (n = 28) ten per cent (based on 233 individuals, n = 335 assays) were selected for genotyping. Fifty-seven birds were tested for the end region of *DRD4* and, when this did not show any variation, we tested nineteen birds for the start region of *DRD4*. An additional twenty-eight birds measured once for exploratory behaviour were included in the *SERT* analysis, resulting in a total sample size of eighty-five. The number of individuals measured for bold behaviour was lower because bold behaviour was not assayed in earlier years.

Blood samples were collected and stored in absolute ethanol. DNA was extracted using either a phenol extraction technique [44] or a salt extraction method [25]. SNP genotyping was performed based on the PCR methods of Kenta *et al*. [45]; modifications included 4 μl of Qiagen PCR master mix, 1 μl of each forward and reverse primer at 5 μM, 1 μl of DNA (~10 ng/μl) and 3 μl of ddH_2_O per PCR reaction. The Sanger sequencing protocol was modified using 1/8 of the BigDye® Terminator Cycle Sequencing reagents 3.1 (Applied Biosystems). Sets of primers were used for sequencing on the ABI3730 sequencer.

Sequences were aligned in CodonCode Aligner 5.1.4 (Codon Code Corporation, www.codoncode.com) and visually examined for polymorphisms. Note that 3 base pairs at position 80 were missing in two samples. Construction of haplotypes followed in DNAsp 5.10.1 [46]; sequences are provided in S3 Table.

### Statistical analyses

Statistical analyses were performed in R 3.0.1 [47] to analyse the *SERT* polymorphisms. We used Haldane’s exact test from the Hardy-Weinberg package 1.5.2 [48] to assess whether SNP frequencies deviated from Hardy-Weinberg equilibrium (HWE).

A generalised linear mixed model, GLMM, was run in lme4 1.1–5 [49] using the function glmer with a Poisson error distribution and log link, adjusted for over-dispersion [50]. We ran both overdominant and additive models to investigate different SNP/haplotype effects on the assayed personality traits. For the SNP analyses, the independent variables were each SNP (*SNP147*, *SNP209*, *SNP446*, *SNP467*) as a factor for the overdominant model (3 levels i.e. Aa, aa, AA) and SNP as a continuous variable for the additive model (0, 1, 2 copies of SNP). For the haplotype analyses, the independent variables were each haplotype as a factor for the overdominant model (5 haplotypes with 0, 1, 2 copies of a haplotype) and haplotype as a continuous variable for the additive model (0, 1 or 2 copies of the haplotype). Assay number, see S4 Table for repeat assay number, [51], social status, subordinate or dominant [52], sex [53] and age [54] have been shown to correlate with personality so were included as fixed effects. Age was mean centred and divided by two standard deviations [55] and, to account for non-linear relationships it was included as a quadratic effect. Bird identity and observer identity were included as random effects, as the analyses included birds with repeat personality assays and measurements by more than one observer. The significance of SNP/haplotypes was assessed using a likelihood ratio test (LRT) with a null model excluding the SNP/haplotype effect. The p-values for each model were corrected for multiple testing with false discovery rate [56]. Including personality tent colour did not alter the results.

## Results

*DRD4* was monomorphic, however four SNPs were identified in *SERT* at *SNP147*, *SNP209*, *SNP446* and *SNP467* in the non-coding end region. None of the four SNPs deviated from Hardy-Weinberg equilibrium (S5 Table). The five haplotype sequences clustered with the blackbird and great tit *SERT* exon one sequences, and the mRNA sequences for the chicken, collared flycatcher and zebra finch clustered together (S1 Fig).

**Fig 2:**
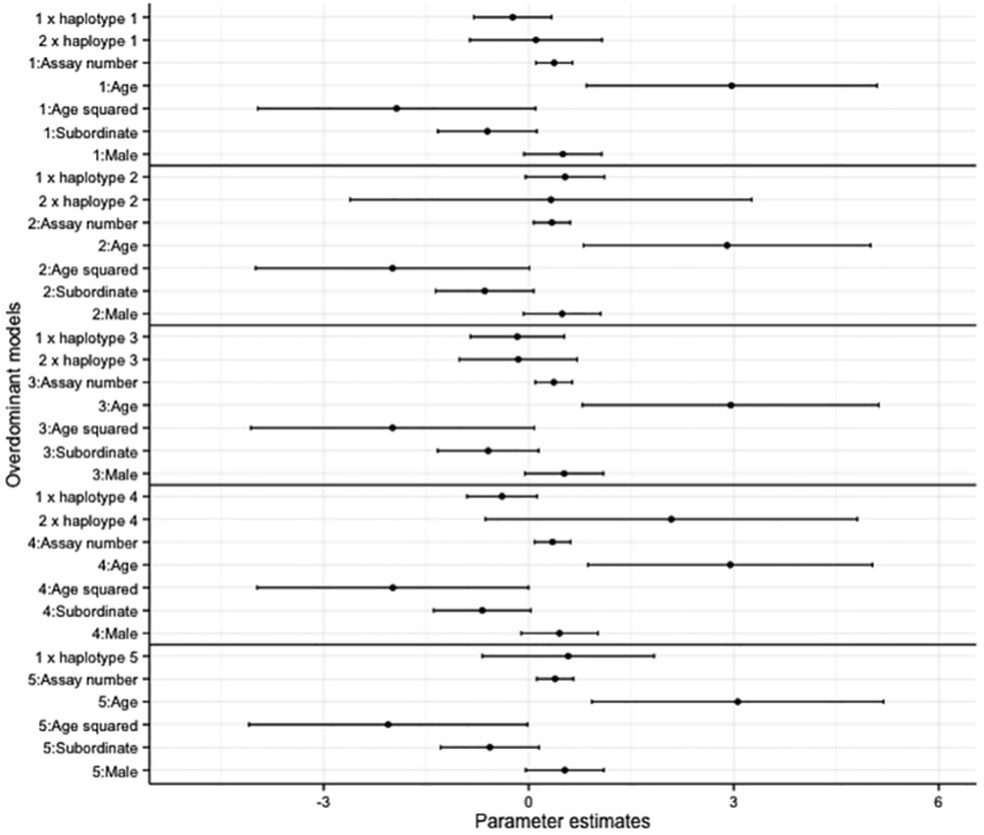
The coefficients and associated 95% confidence intervals (CI) of the overdominant haplotype models for exploratory behaviour. The models are relative to individuals with no copies of the haplotype, subordinate is relative to dominant, male is relative to female.

**Fig 3:**
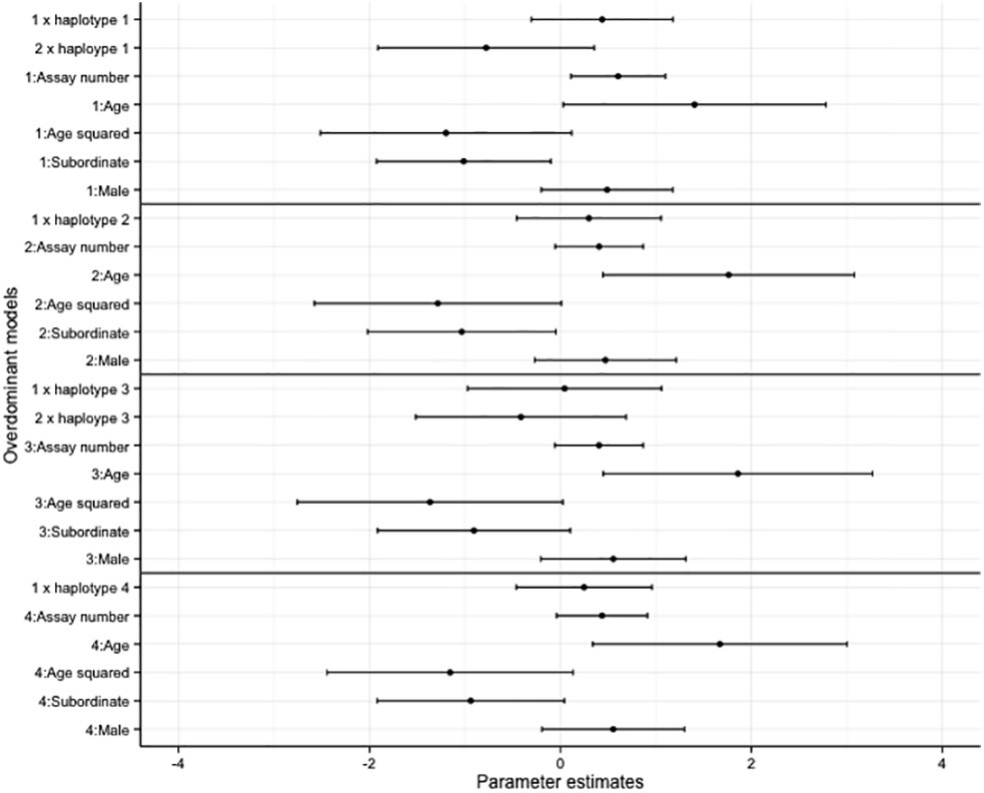
The coefficients and associated 95% confidence intervals of the overdominant haplotype models for bold behaviour. The models are relative to individuals with no copies of the haplotype, subordinate is relative to dominant, male is relative to female.

**Fig 4:**
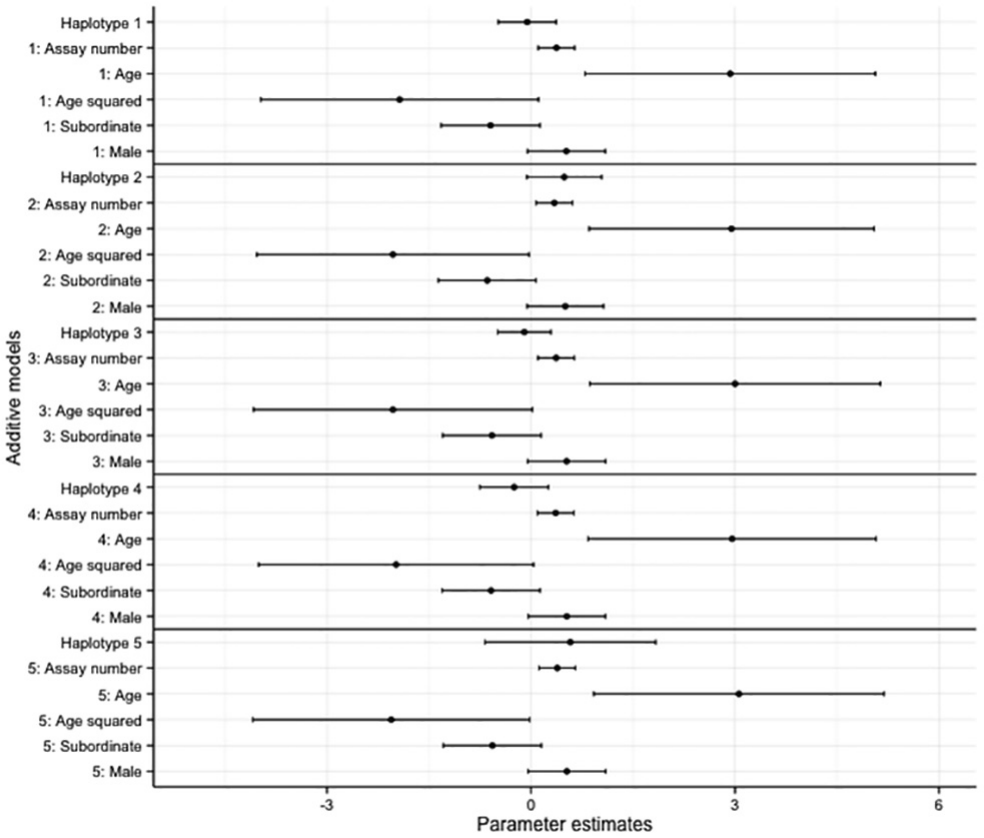
The coefficients and associated 95% confidence intervals of the additive haplotype models for exploratory behaviour. The models are relative to individuals with no copies of the haplotype, subordinate is relative to dominant, male is relative to female.

**Fig 5:**
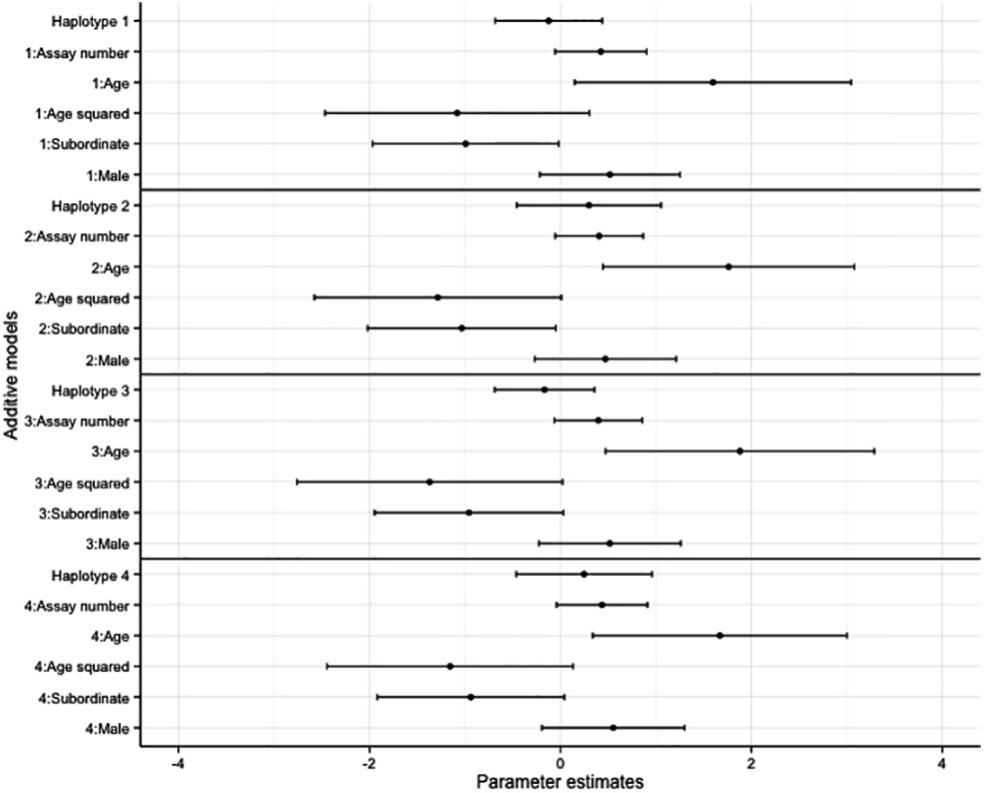
The coefficients and associated 95% confidence intervals of the additive haplotype models for bold behaviour. The models are relative to individuals with no copies of the haplotype, subordinate is relative to dominant, male is relative to female.

Overall, there was no effect of haplotype on bold and exploratory behaviours in the overdominant and additive models (Figs 2–5, accompanying LRT values can be found in S6 and S7 Tables). Similarly no SNP effect was seen in the overdominant and additive models for bold and exploratory behaviour (S2–S5 Figs, accompanying LRT values can be found in S6 and S7 Tables). There was a positive correlation with age for exploratory behaviour and bold behaviour in all models (haplotype models Figs 2–5; SNP models S2–S5 Figs). Dominant individuals were bolder than subordinates in the additive and overdominant models for haplotypes one and two. Individuals also became bolder with increasing assay number in the overdominant model for haplotype one (Fig 3).

## Discussion

Identifying the genetic basis of personality can greatly further our understanding of why between-individual differences in behaviour persist in populations. Despite *DRD4* polymorphisms being previously associated with exploratory behaviour [18], the portion of this locus that we sequenced was monomorphic in our study population. Having sequenced both the start and end regions, which represents 11% of the length of *DRD4*, from the regions that vary in passerines, it is unlikely that we missed variation in this gene, but without sequencing the whole gene we cannot rule this out. Polymorphisms were, however, found in *SERT*, but these did not correlate with variation in exploratory behaviour or bold behaviour.

Despite our null result, we cannot rule out induced and inherited changes in a gene's expression (known as epigenesis) influenced by factors such as age or the external environment [57]. Blue tit nestlings showed genetic correlations between two personality traits (aggression and stress) that disappeared in adulthood. This was thought to be due to a change in the expression of the genes determining the traits over development [58], highlighting the importance of studying personality longitudinally [1]. Furthermore, in wild great tits an association between exploratory behaviour and *DRD4* genotype was detected in one of the four tested populations. One of the potential reasons given for this result was environmental differences between populations modifying the genetic effects [18]. In our study species, an additional four populations have been founded from the sequenced population; the first translocation was in 1988 and the most recent was in 2011 [59]. It would be of interest to investigate whether genotype by environment interactions occur within or between the five island populations.

An association between candidate genes and personality does not always imply a direct functional effect [60]. For example LD was found between *DRD4* polymorphisms and polymorphisms in the neighbouring *DEAF1*, involved in the regulation of the serotonergic system, in chickens [61]. Although LD is not known in the Cousin population of the Seychelles warbler it is expected to be high. The population has experienced a relatively recent bottleneck around 120–250 years ago (33–64 generations), reducing the population to around 29–75 individuals [62,63], and consequently reducing genetic diversity by 25% and heterozygosity by 19% [63]. Therefore, the number of recombination events since the bottleneck will be small [60,64]. Additionally, it is a small population with a very low dispersal rate between islands [60,65]. High LD increases the power to detect a correlation between the *SERT* polymorphisms, and bold behaviour and exploratory behaviour, because of higher linkage with other personality related genes close by.

Being unable to identify the genes underlying focal traits is one disadvantage of the candidate gene approach. Additionally, the candidate gene approach is often biased towards genes with large effect sizes [66] and this may be amplified by the publication of mainly positive results [8]. As yet, it is unknown how many studies have found a null result and not published the findings. This is why it is important to publish all studies, including studies with null results like ours, to allow for more representative meta-analyses to be conducted. Nevertheless, we chose the candidate gene approach to maximise the likelihood of detecting ageing effects while minimising the chance of type I and II errors [67].

A future direction could be to employ a genome-wide study to look for signatures of selection on personality. The bottlenecked past of our study species may have left signatures of selection at other putatively adaptive relevant loci that genome-wide scans could detect. A candidate gene approach could then follow at these loci to investigate their association with personality [66,68]. However, it is then necessary to rule out the possibility of pleiotropy, correlated selection or transgenerational epigenetic effects [69]. Alternatively, genome-wide study could look at the partitioning of genetic variance, which would facilitate detection of relevant genes located in genomic regions with small effect sizes [70].

## Conclusion

Understanding the molecular genetic basis of personality can ultimately help to explain why behavioural differences between individuals occur in populations. Studies in wild populations that experience natural selective pressures will allow us to address these questions. We found no association between these behaviours and variation in the candidate genes tested in our study population. Future work should account for age or environment effects on *SERT* variants and investigate underrepresented candidate genes that may have an additive or pleiotropic effect on personality. We emphasise the importance of studying personality throughout development in a controlled longitudinal study and the need for the publication of null findings to aid future meta-analyses on personality candidate genes.

## Supporting Information

S1 Fig Neighbour-joining phylogenetic tree of avian *SERT* chromosome 19 sequences constructed in Mega 5.2 [39]. Numbers at branching points represent bootstrap values inferred from 5000 replicates. The horizontal scale bar indicates 0.1 nucleotide substitutions per site.
(TIFF)

S2 Fig The coefficients and associated 95% confidence intervals (CI) of the overdominant SNP models for exploratory behaviour. The models are relative to homozygotes with the G/C/A SNP, subordinate is relative to dominant, male is relative to female.
(TIFF)

S3 Fig The coefficients and associated 95% confidence intervals (CI) of the overdominant SNP models for bold behaviour. The models are relative to homozygotes with the C/A SNP, subordinate is relative to dominant, male is relative to female.
(TIFF)

S4 Fig The coefficients and associated 95% confidence intervals (CI) of the additive SNP models for exploratory behaviour. The models are relative to individuals with no copies of the SNP, subordinate is relative to dominant, male is relative to female.
(TIFF)

S5 Fig The coefficients and associated 95% confidence intervals (CI) of the additive SNP models for bold behaviour. The models are relative to individuals with no copies of the SNP, subordinate is relative to dominant, male is relative to female.
(TIFF)

S1 Table Breakpoint analysis. A linear mixed model (LMM) was run in lme4 1.1–5 [49] with exploration score as the dependant variable, minute and the breakpoint as independent variables and bird identity as a random effect. The model had random slope variances and a random intercept variance for a break point at minute 10. The R function optimize was used to estimate the breakpoint.
(TIFF)

S2 Table Power analysis using R package pwr 1.1–2 [43] and the effect size from the Westerheide population [18]. U is the degrees of freedom in the numerator. The sample size (N) is calculated by adding the degrees of freedom in the denominator, U and one.
(TIFF)

S3 Table The sequences of the five *SERT* haplotypes. Bold nucleotides indicate the SNPs.
(TIFF)

S4 Table Number of Seychelles warblers with repeat assays for exploratory and bold behaviour. (TIFF)

S5 Table Nucleotide change and p-values of Haldane’s exact test for each *SERT* SNP. (TIFF)

S6 Table Likelihood ratio test results for exploratory behaviour in the overdominant and additive models using SNPs and haplotypes. False Discovery Rate (FDR) p values control for running four SNP models and five haplotype models with alpha set at 0.05. d.f. = degrees of freedom.
(TIFF)

S7 Table Likelihood ratio test results for bold behaviour in the overdominant and additive models using SNPs and haplotypes. False Discovery Rate (FDR) p values control for running four SNP models and four haplotype models with alpha set at 0.05. d.f. = degrees of freedom.
(TIFF)
