# Supplementary figures and images for: No Association between Personality and Candidate Gene Polymorphisms in a Wild Bird Population
Source: PLoS One. 2015 Oct 16;10(10):e0138439. doi: 10.1371/journal.pone.0138439 (PMC4608812; doi:10.1371/journal.pone.0138439)

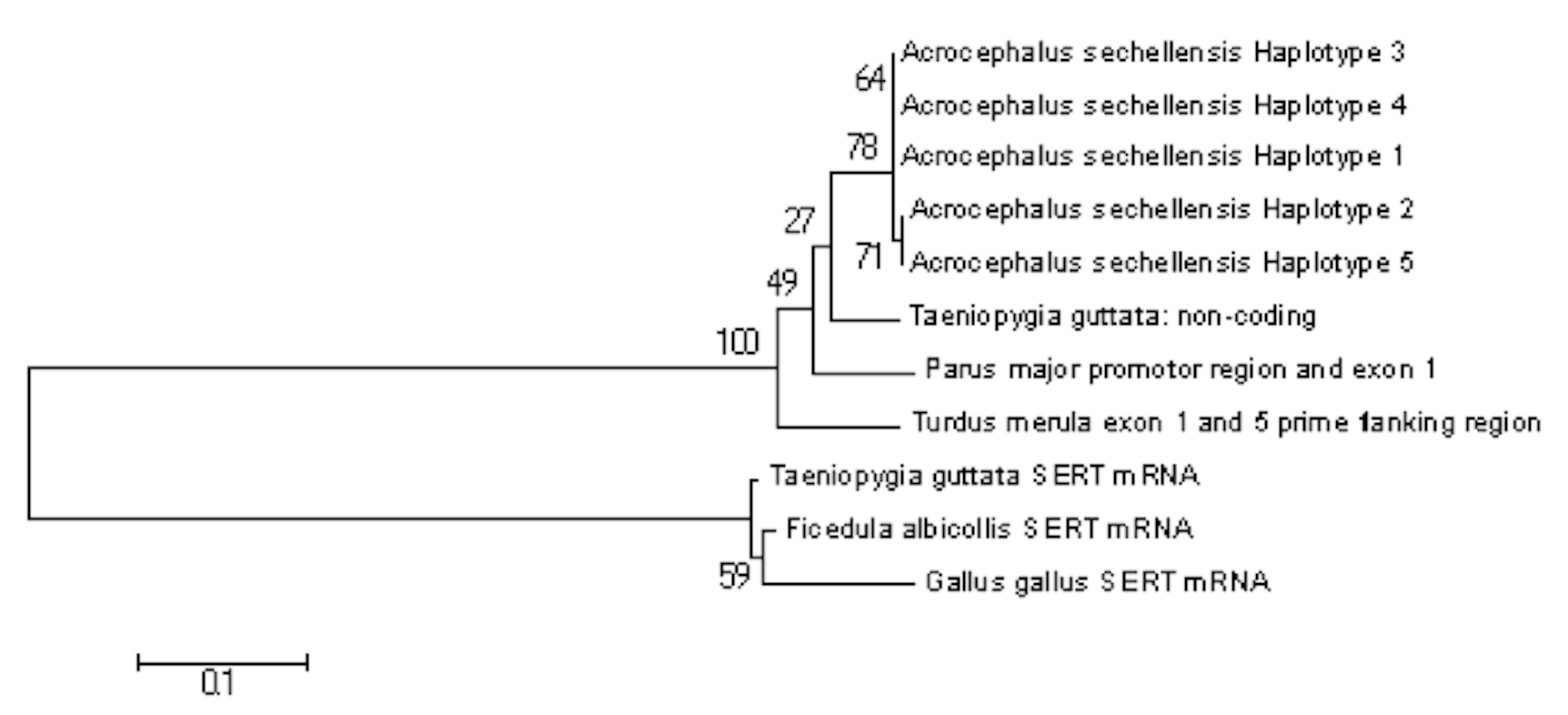

Supplement: S1 Fig — Numbers at branching points represent bootstrap values inferred from 5000 replicates. The horizontal scale bar indicates 0.1 nucleotide substitutions per site. (TIFF) [file pone.0138439.s001.tiff]

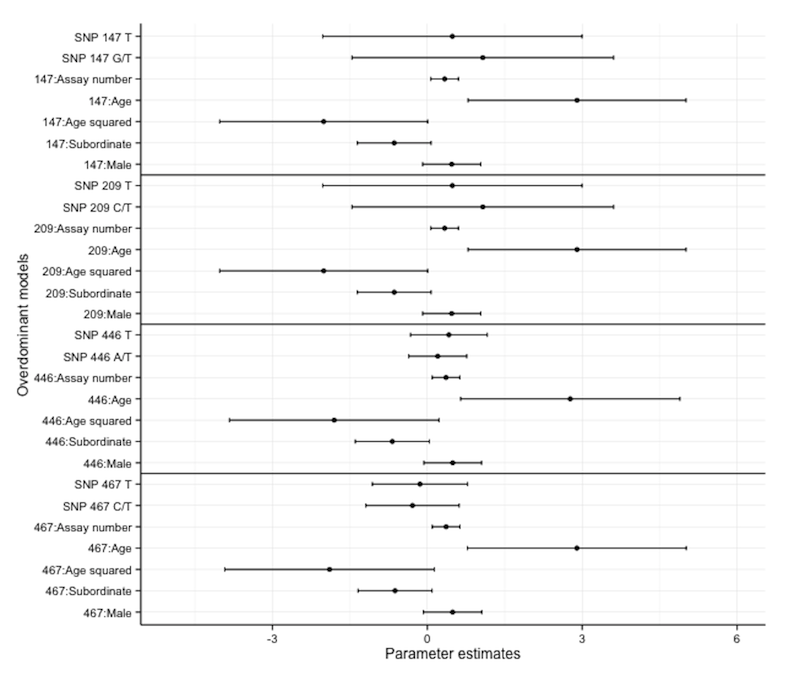

Supplement: S2 Fig — The models are relative to homozygotes with the G/C/A SNP, subordinate is relative to dominant, male is relative to female. (TIFF) [file pone.0138439.s002.tiff]

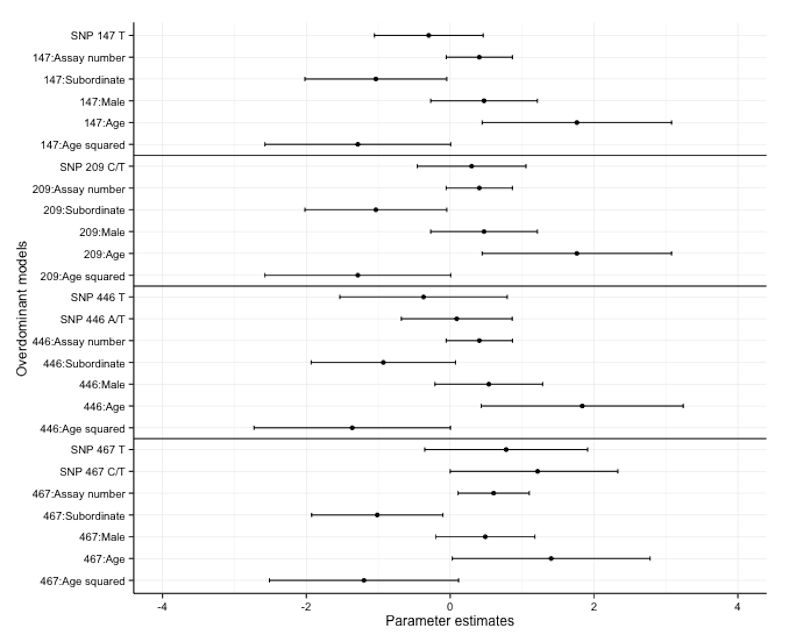

Supplement: S3 Fig — The models are relative to homozygotes with the C/A SNP, subordinate is relative to dominant, male is relative to female. (TIFF) [file pone.0138439.s003.tiff]

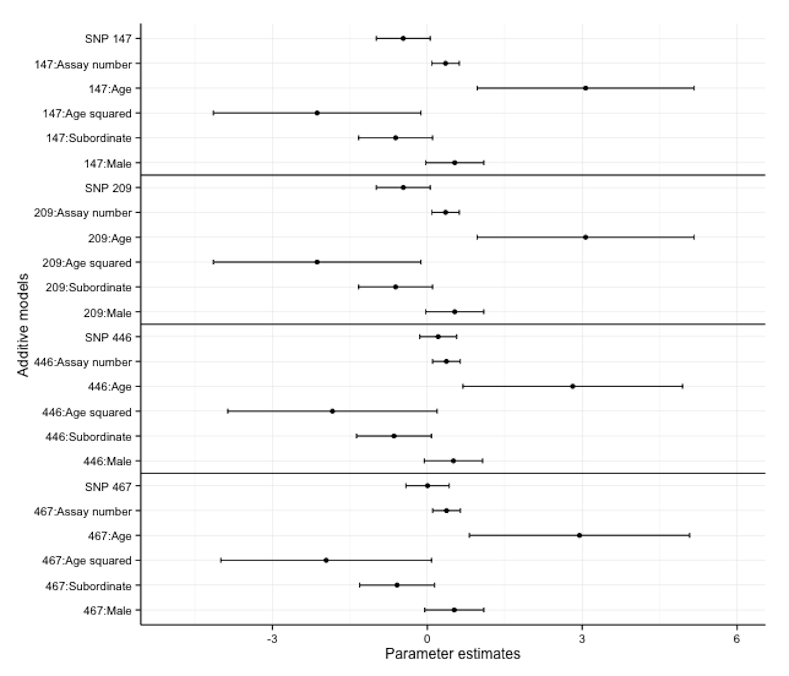

Supplement: S4 Fig — The models are relative to individuals with no copies of the SNP, subordinate is relative to dominant, male is relative to female. (TIFF) [file pone.0138439.s004.tiff]

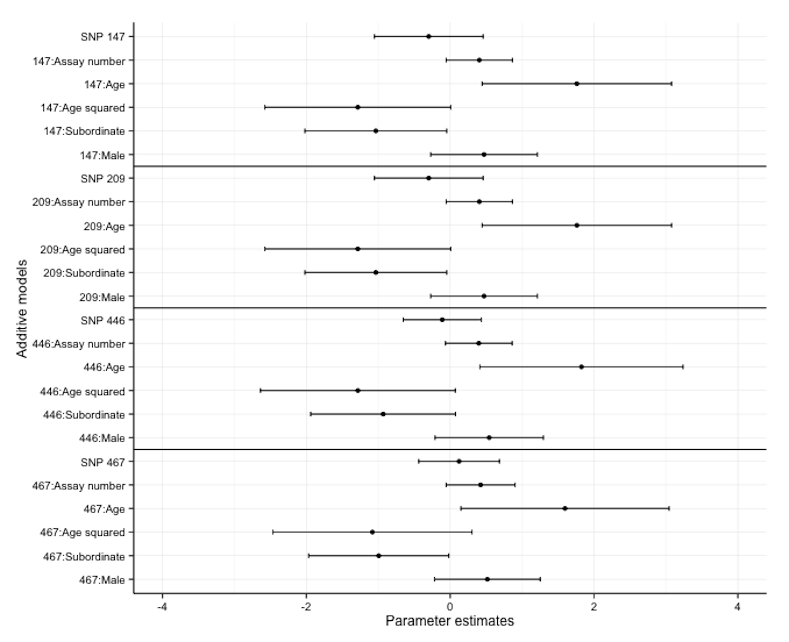

Supplement: S5 Fig — The models are relative to individuals with no copies of the SNP, subordinate is relative to dominant, male is relative to female. (TIFF) [file pone.0138439.s005.tiff]

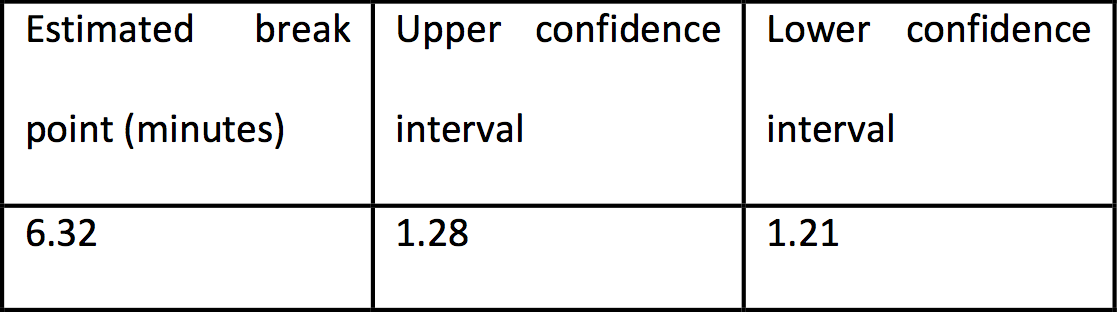

Supplement: S1 Table — A linear mixed model (LMM) was run in lme4 1.1–5 [49] with exploration score as the dependant variable, minute and the breakpoint as independent variables and bird identity as a random effect. The model had random slope variances and a random intercept variance for a break point at minute 10. The R function optimize was used to estimate the breakpoint. (TIFF) [file pone.0138439.s006.tiff]

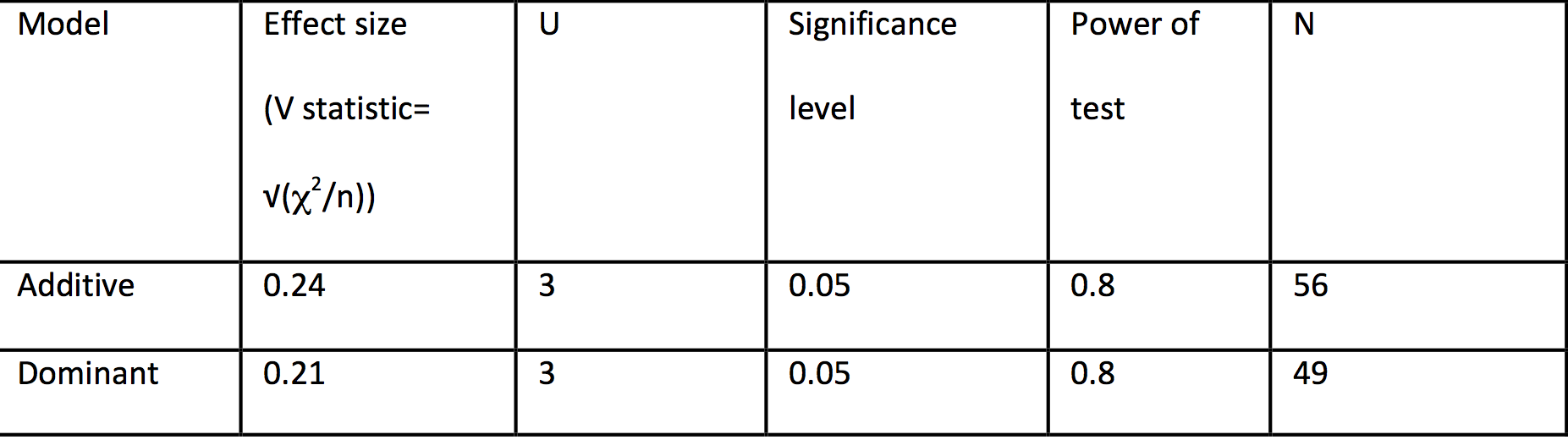

Supplement: S2 Table — U is the degrees of freedom in the numerator. The sample size (N) is calculated by adding the degrees of freedom in the denominator, U and one. (TIFF) [file pone.0138439.s007.tiff]

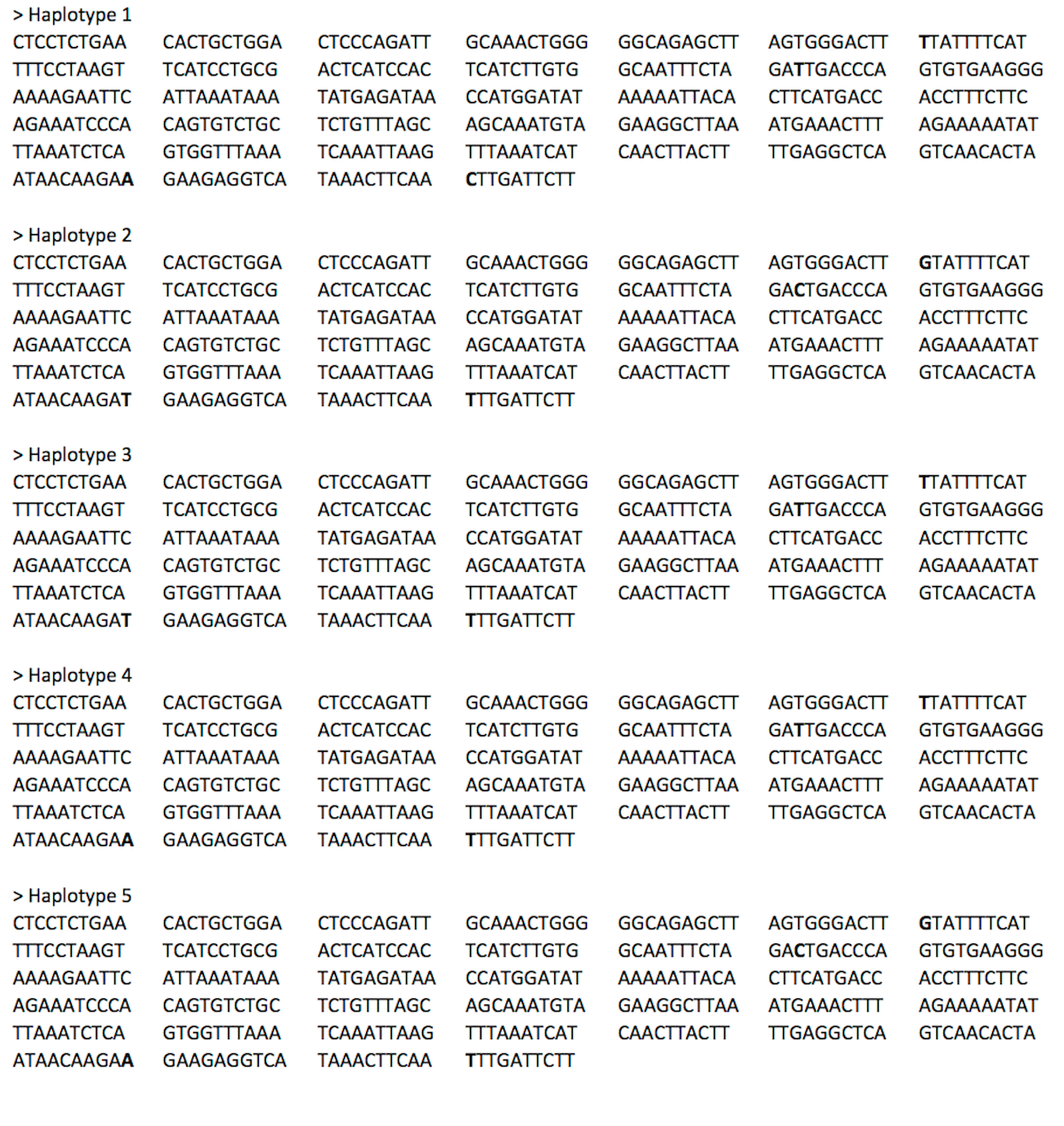

Supplement: S3 Table — Bold nucleotides indicate the SNPs. (TIFF) [file pone.0138439.s008.tiff]

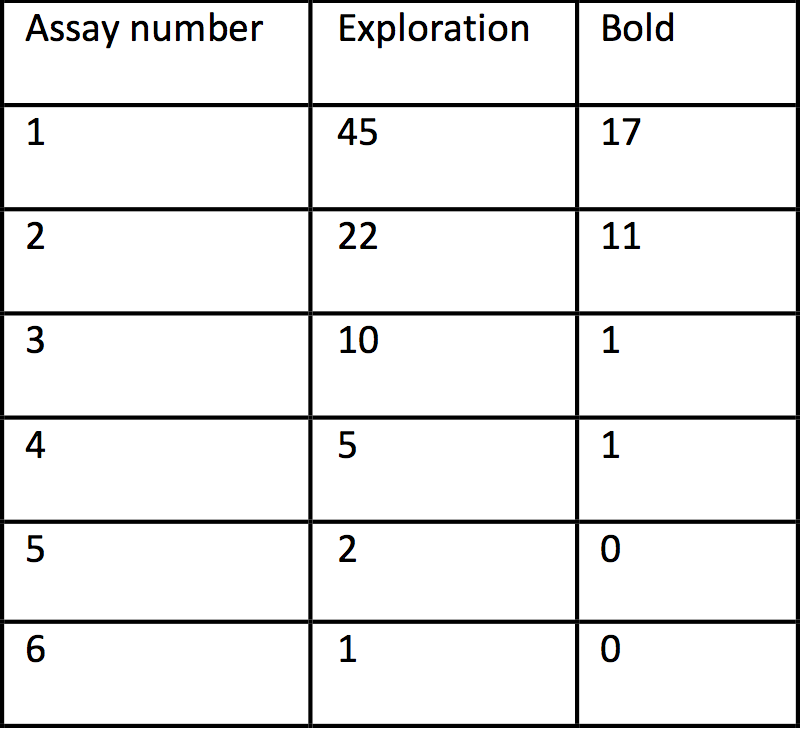

Supplement: S4 Table — (TIFF) [file pone.0138439.s009.tiff]

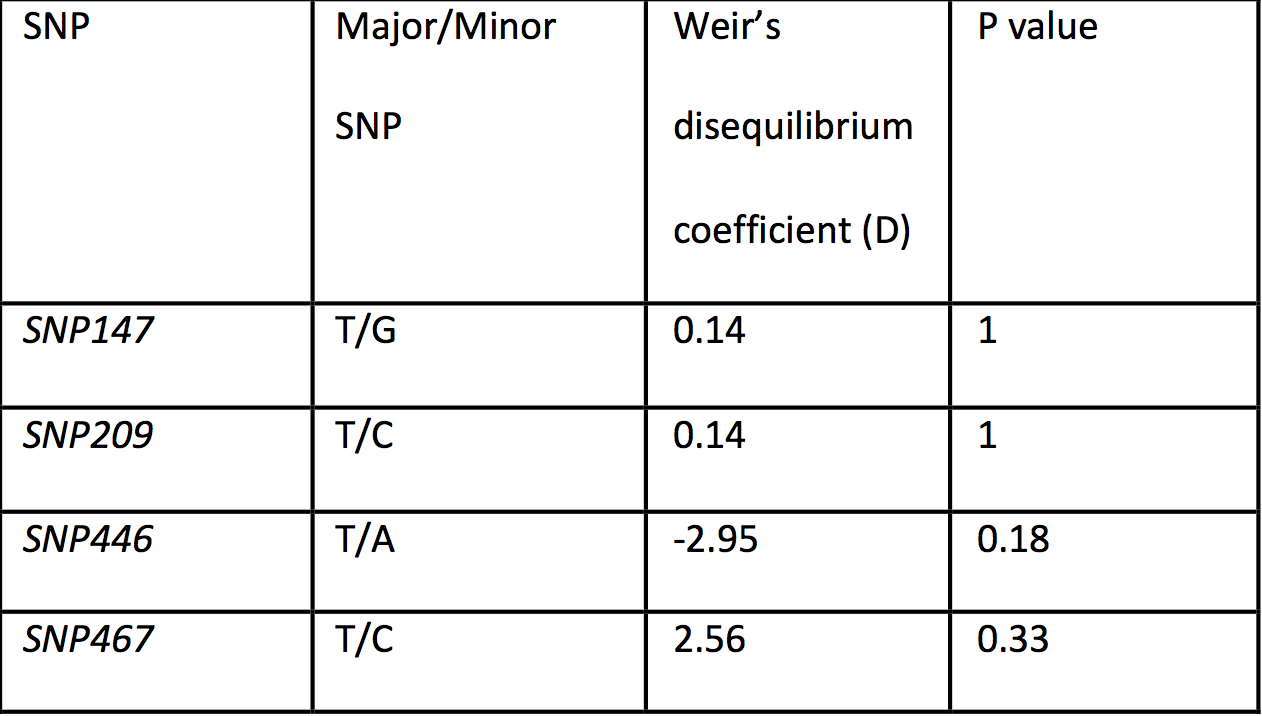

Supplement: S5 Table — (TIFF) [file pone.0138439.s010.tiff]

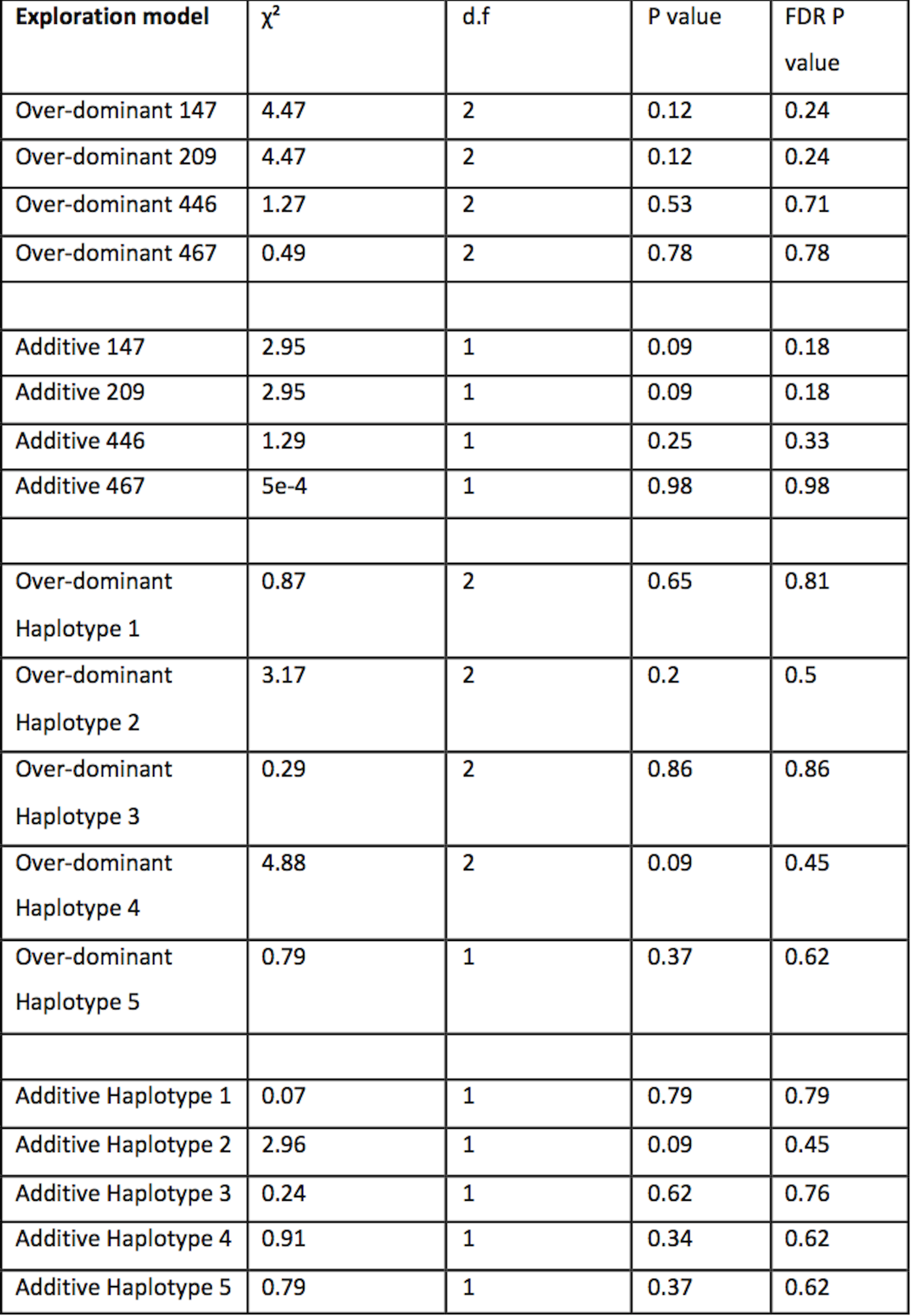

Supplement: S6 Table — False Discovery Rate (FDR) p values control for running four SNP models and five haplotype models with alpha set at 0.05. d.f. = degrees of freedom. (TIFF) [file pone.0138439.s011.tiff]

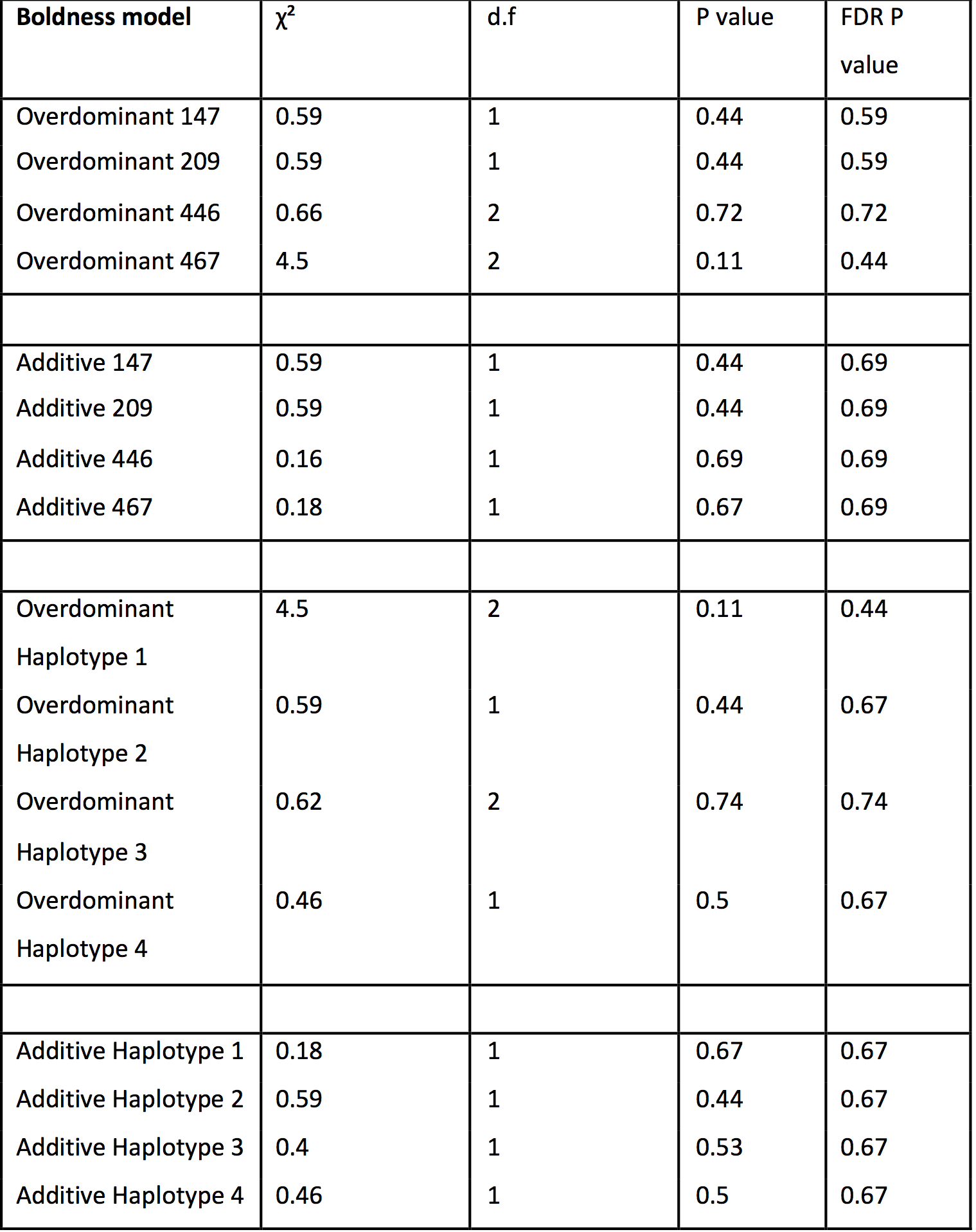

Supplement: S7 Table — False Discovery Rate (FDR) p values control for running four SNP models and four haplotype models with alpha set at 0.05. d.f. = degrees of freedom. (TIFF) [file pone.0138439.s012.tiff]
